# Artificial intelligence in periodontics and peri-implant medicine: from diagnosis to precision care

**DOI:** 10.3389/fdmed.2026.1816844

**Published:** 2026-05-04

**Authors:** Poojita Yadavalli, Saravanan Sampoornam Pape Reddy, L. Kesavalu

**Affiliations:** 1 College of Dentistry, University of Florida, Gainesville, FL, United States; 2 Army Dental Corps, Bhopal, India; 3 Department of Periodontology, Adjunct Professor of Oral Biology, College of Dentistry, University of Florida, Gainesville, FL, United States

**Keywords:** artificial intelligence, implantology, machine learning, neural networks, peri-implant medicine, periodontics

## Abstract

**Background:**

Artificial intelligence (AI) has substantial potential to advance periodontal and peri- implant care through novel diagnostic, prognostic, and therapeutic opportunities. Nevertheless, the evidence is scattered over several fields.

**Objective:**

To provide a narrative synthesis of the recent evidence regarding applications of AI to periodontology and peri-implant medicine specifically diagnostic imaging, disease prediction, biomarker discovery, peri-implant disease assessment and clinical decision support.

**Methods:**

A systematic search of PubMed, MEDLINE, Scopus, and Embase for studies from August 2020 to August 2025 was performed. We included only original research articles on AI applications in periodontal or peri-implant settings. After screening the identified records and conducting full-text assessments, a total of 32 studies were included based on established inclusion criteria.

**Results:**

AI showed high diagnostic performance at imaging-based applications, with accuracy from 0.85 to 0.98 reported for the detection and quantification of alveolar bone loss using two- and three-dimensional radiographs. Using this data, machine learning (ML) models were developed for identifying potential molecular biomarkers that could support disease prediction and gain insight into biological processes. AI-aided immune profiling and risk stratification models demonstrated robust predictive capabilities in peri-implant maintenance. mHealth is one of the types of AI-based digital health devices and has succeeded in enhancing patient compliance and periodontal prognosis.

**Conclusions:**

AI has considerable promise to impact non-invasive periodontal and peri-implant diagnostics, risk-assessment and enhanced treatment-planning through advanced imaging analysis, molecular profiling as well as patient-centred digital tools. These need to be further validated, standardized, and integrated into clinical care before widespread uptake.

## 1 Introduction

The diagnosis of periodontal diseases (PD), including periodontitis and peri-implantitis (PI), remains challenging due to the progressive destruction of tooth- or implant-supporting tissues (1). Clinical assessment relies on labour-intensive procedures - probing pocket depth measurement, evaluation of bleeding on probing, and radiographic interpretation that are influenced by clinician experience and require consistent adherence to the 2018 periodontal classification guidelines established by the American Academy of Periodontology (AAP) and the European Federation of Periodontology (EFP) (2, 3). Given the high global prevalence of PD, there is a critical need for rapid, reliable, and standardized diagnostic approaches (4). Artificial intelligence (AI) has emerged as a transformative tool in dentistry, enabling automation of complex diagnostic tasks while reducing operator-dependent variability. AI-driven systems have demonstrated strong performance in early disease detection, assessment of disease progression, and quantification of alveolar bone loss (ABL) (5, 6). Beyond imaging, biomolecular studies incorporating machine learning have enabled the identification of key molecular biomarkers, improving understanding of host responses to microbial toxins (6, 7). These approaches also facilitate antimicrobial resistance (AMR) prediction and elucidate associations between PD and systemic conditions, including cardiovascular, metabolic, and malignant diseases (7, 8).

**Figure 1:**
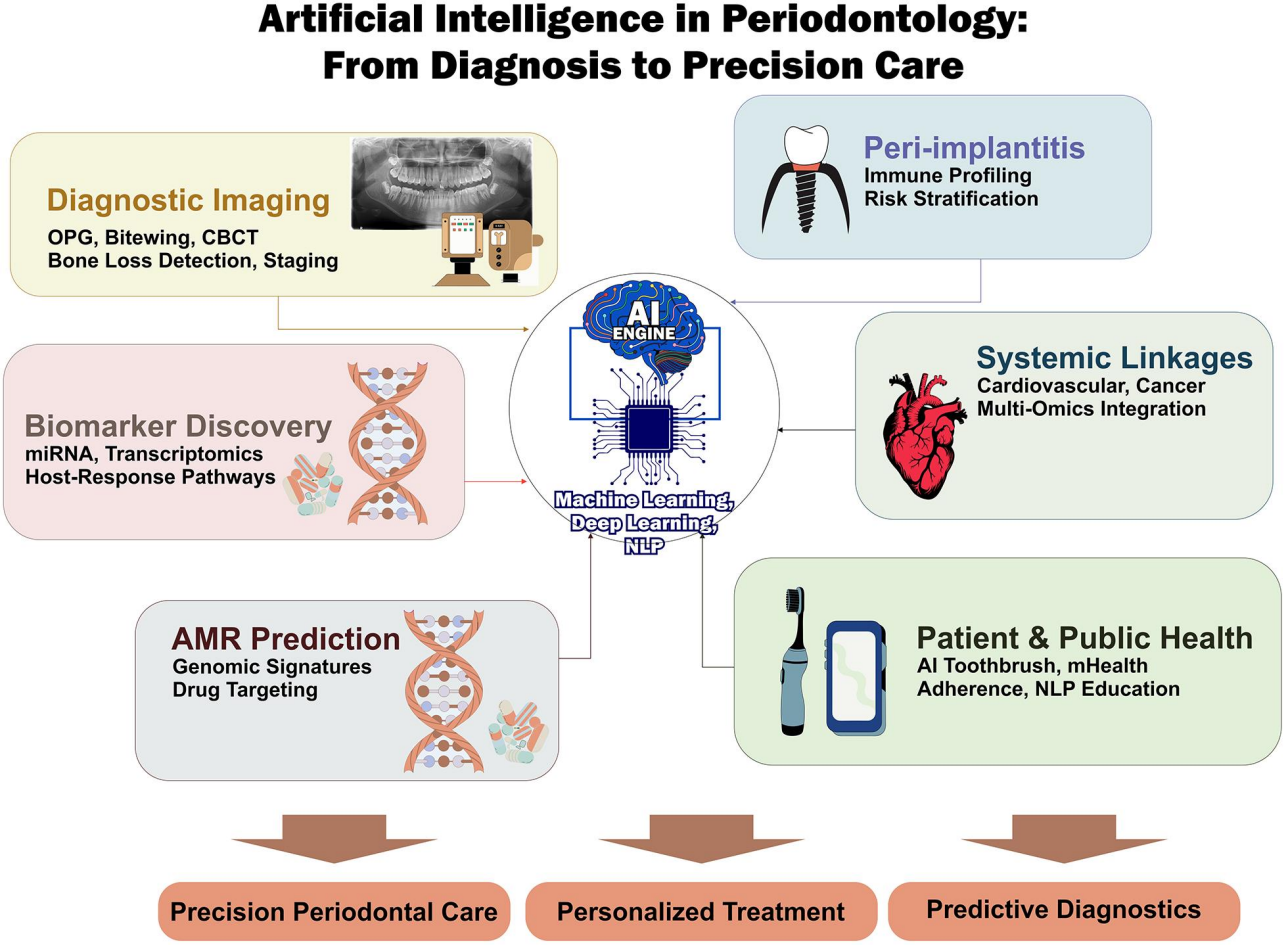
Conceptual framework of artificial intelligence applications in periodontology. The schematic illustrates key domains of AI integration, including diagnostic imaging (2D and 3D radiographs), peri-implant disease profiling, biomarker discovery, systemic disease linkage, antimicrobial resistance prediction, and patient-centred digital health interventions. These domains converge toward precision periodontal care, personalized treatment strategies, and predictive diagnostics.

Despite the rapid expansion of AI applications in dentistry, its integration into Periodontology and Peri-implant medicine remains fragmented, with evidence dispersed across imaging, molecular biology, and clinical decision-support domains. Given the shared pathogenic mechanisms and overlapping clinical management strategies between periodontal and peri-implant diseases, AI-driven approaches in implant diagnostics and guided surgery were also included to provide a comprehensive perspective on precision oral healthcare. A structured synthesis is required to consolidate these developments and critically evaluate their translational relevance. This review is therefore intended to provide clinicians and researchers with an integrated understanding of AI-driven innovations in Periodontology, with particular emphasis on their potential to improve diagnostic precision, risk stratification, and personalized treatment planning. This review addresses the following research question: How is AI currently applied across diagnostic, prognostic, and therapeutic domains in Periodontology and Peri-implant medicine, and what is the strength of evidence supporting its clinical utility? Accordingly, the aim of this narrative review was to systematically synthesize recent evidence on AI applications in Periodontology and Peri-implant medicine, with a focused evaluation of diagnostic performance, biological insights, and clinical translation potential. A conceptual overview of the major applications of AI in Periodontology and Peri-implant medicine is illustrated in Figure 1.

## 2 Material and methods

This narrative review was developed in adherence to SANRA guidelines based on a detailed systematic literature search conducted across several databases, including PubMed, Scopus, MEDLINE, and Embase. The BOOLEAN search strategy utilized was AI ‘AND' Periodontics, Periodontology ‘AND' Artificial Intelligence, Periodontology ‘AND' neural networks, AI ‘AND' Machine Learning, and AI ‘AND' Periodontal diseases ‘OR' Periodontal therapy'. The review covered studies published between August 2020 to August 2025. Only original research articles that explored the application of AI in periodontal diagnosis and/or treatment were considered. Studies were excluded if they were not published in English or applied AI for purposes outside the domains of periodontology or implantology. The initial screening was conducted by the first author (P.Y.), focusing on titles and abstracts using the pre-defined MeSH terms and BOOLEAN strategy. Articles deemed relevant underwent a full-text assessment by two blind authors (P.Y. & S.S.P.R). From the initial 1,400 search results, 320 were excluded during the title/abstract screening for failing to meet the inclusion criteria. If there was any disagreement, the consensus arrived after discussion with the expert reviewer (L.K). The summary of the search strategy is presented in Table 1. After full-text evaluation, 32 studies remained for inclusion in this review. A summary of the included studies is presented in Table 2.

**Table 1:** Summary of search strategy.

Item	Specification
Date of search	10 Aug 2025
Databases searched	PubMed, Scopus, MEDLINE, Embase
Search terms (MeSH)	“Periodontics AND AI”, “Periodontology AND Artificial Intelligence”, “Periodontology AND neural networks”, “*AI AND Machine Learning*”, “AI AND periodontal diseases and periodontal therapy”
Timeframe	2020–2025
Inclusion criteria	Original articles applying AI to periodontal/peri-implant diagnosis/treatment/prognosis
Exclusion criteria	Non-English publications, AI applied outside Periodontology/Implantology
Selection process	Initial screening by a single author (P.Y.), final selection after independent blinded review by two authors (P.Y. & S.S.P.R.), and consensus was achieved. If there were any conflicts between the two authors, the third author (L.K) resolved them.

**Table 2:** Summary of included studies evaluating artificial intelligence applications in periodontology and peri-implant medicine.

Author (Year)	Study Type	AI Model	Application Domain	Dataset	Sample Size	Key Outcome
Alotaibi et al. (2022) (5)	Retrospective	CNN	ABL detection	Radiographs	1,724 images	Accuracy ∼0.90 for bone loss detection
Ossowska et al. (2022) (6)	Observational	Neural networks	Disease progression	Clinical data	110 patients	Effective prediction of periodontitis progression
Antony et al. (2023)	Experimental	ML (CLDB)	Periodontitis staging	OPG images	200 images	Accurate stage classification
Kong et al. (2023) (10)	Experimental	CNN (PDCNN)	ABL detection & staging	OPG dataset	1,747 images	Automated detection with high precision
Kurt-Bayrakdar et al. (2024) (11)	Retrospective	Deep learning	Pattern recognition	Panoramic radiographs	1,121 images	Improved defect classification
Cerda et al. (2024) (12)	Comparative	ML models	Bone loss measurement	Clinical imaging	2,010 images	Comparable to specialists
Lee et al. (2025) (13)	Clinical evaluation	AI software	ABL quantification	Clinical dataset	550 images, and 56 dental proffesionals	High agreement with clinicians
Huang & Huang (2024) (14)	Experimental	CNN + SVM	Diagnosis classification	Clinical data	12,774 images	Reliable classification of disease
Tian et al. (2025) (8)	Validation	ML (Polyfit)	ABL quantification	Radiographs	290 patients	Improved measurement accuracy
Kurt-Bayrakdar et al. (2025) (15)	Experimental	Deep learning	CBCT segmentation	CBCT scans	502 patients	Accuracy up to ∼0.99
Wang et al. (2021) (18)	Observational	ML (FARDEEP)	Peri-implantitis	Transcriptomics	24 patients	AUC >0.85 risk prediction
Rakic et al. (2023) (17)	Systematic review	—	Peri-implantitis epidemiology	Literature	29 articles	High prevalence variability
Gupta et al. (2025)	Experimental	AI-guided planning	Implant surgery	Clinical cases	75 models	Improved surgical precision
Li et al. (2024) (33)	RCT	AI-enabled device	Patient adherence	Clinical patients	NR	Improved plaque control
Sabri et al. (2025) (36)	Comparative	LLMs	Education	Exam datasets	1,312 questions	High AI performance
Tastan Eroglu et al. (2024) (35)	Experimental	ChatGPT	Diagnosis classification	Clinical scenarios	200 Digital data	Moderate–high accuracy
Shyamsukha et al. (2025) (37)	Observational	LLM	Education support	Students	10 questions, and 30 periodontists	Useful adjunct tool
Yadalam et al. (2024)	Experimental	Deep learning	miRNA analysis	Cell models	NR	Identified biomarkers
Yadalam et al. (2025a) (30)	Experimental	ML	AMR prediction	Genomic data	685 Protein sequences	High predictive accuracy
Yadalam et al. (2025b) (31)	Experimental	CNN	AMR classification	Protein sequences	304 resistant sequences and 261 regular sequences	Classification of resistance
Yadalam et al. (2025c) (32)	Experimental	Autoencoder	Keratinocyte profiling	scRNA-seq	4 samples	Identified 28 subpopulations
Wu et al. (2025) (22)	Integrative analysis	ML	Periodontitis–cancer link	RNA-seq	310 gingival papillae	Identified hub genes
Joyson et al. (2025) (23)	Experimental	ANN	COVID–periodontitis link	Molecular data	Top 500 genes	Shared inflammatory pathways
Aravindraja et al. (2023a) (24)	Experimental	ML	miRNA profiling after infection with *Tanerella Forsythia*	Animal model	10	Dysregulated miRNA signatures
Aravindraja et al. (2024) (26)	Experimental	ML	Microbial interaction after infection with *Streptococcus gordonii*	Animal model	10	Immune response modulation
Jeepipalli et al. (2025) (29)	Experimental	ML	Microbial–cancer link after infection with *Fusobacterium nucleatum*	Molecular data	10	Oncogenic miRNA induction
Angjelova et al. (2024) (21)	Clinical	ML	CVD linkage	Clinical dataset	1,497 records	Endothelial dysfunction link
Yan et al. (2025)	Observational	ML	Systemic association	Clinical data	667 cross sectional cohort	Strong association patterns
Chen et al. (2025)	Experimental	AI foundation model	Histopathology	Multi-omics	2.2 mil paired tissue, 32 organs	Integrated imaging-genomics
Sowmya et al. (2025)	Experimental	Gradient boosting	Morphology analysis	Imaging data	200 CBCT, 200 OPG images	Accurate classification
Yadalam et al. (2025d) (33)	Experimental	Few-shot learning	ABL classification	Radiographs	1,965 images	Improved classification
Yadalam et al. (2025e) (34)	Experimental	Graph neural networks	Drug-gene association	Molecular data	Top 300 enteries	Identified therapeutic targets

Summary of included studies evaluating artificial intelligence applications in periodontology and peri-implant medicine. ABL, alveolar bone loss; CNN, convolutional neural network; ML, machine learning; CBCT, cone-beam computed tomography; ANN, artificial neural network; LLM, large language model; NR, not reported.

## 3 Artificial intelligence for diagnostic imaging in periodontics

AI applications in periodontal imaging have predominantly focused on the detection, quantification, grading, and pattern classification of ABL (5, 9, 10). Radiographic images are typically manually annotated by clinicians to identify areas of bone loss, which are then used to train and validate AI algorithms. Most studies have relied on 2D imaging modalities, including orthopantomograms (OPGs), bitewing radiographs, and periapical images, whereas fewer investigations have incorporated 3D cone-beam computed tomography (CBCT) to enhance diagnostic precision (5, 11). Convolutional neural networks (CNNs) and artificial neural networks (ANNs) remain the most commonly employed deep learning architectures due to their effectiveness in image recognition and semantic segmentation tasks (6, 9).

### 3.1 Two-dimensional radiographs: detection, severity assessment, and quantification

Periapical radiographs provide high-resolution visualization of individual teeth and adjacent periodontal structures, supporting localized assessment of ABL (5). In contrast, OPGs offer lower spatial resolution but capture the entire dentition in a single acquisition, making them particularly suitable for large-scale AI training and population-level screening (10). Across studies, AI-based models demonstrated high diagnostic performance, with reported accuracy ranging from approximately 0.85 to 0.98, sensitivity from 0.80 to 0.96, and specificity from 0.82 to 0.97 in detecting alveolar bone loss on radiographic images (5, 10, 12). These metrics indicate substantial agreement with expert clinician assessments and support the potential of AI as an adjunctive diagnostic tool.

Recent AI-driven advances in OPG analysis include the development of a two-stage Periodontitis Detection Convolutional Neural Network (PDCNN), trained on a publicly available dataset of 1,747 expert-annotated panoramic radiographs, enabling automated detection of ABL, severity grading, and identification of furcation involvement (10). Adaptive Centre Line–Distance Based (CLDB) segmentation methods have further improved mandibular region analysis and facilitated accurate staging of periodontitis (9). Hybrid AI frameworks combining statistical inference, deep CNN refinement, and rule-based algorithms have enabled quantitative ABL measurement as a percentage and disease stage assignment, achieving diagnostic speeds of approximately 0.02 s per tooth—exceeding human performance (12). The Polyfit approach has additionally addressed panoramic image distortion through ratio- and proportion-based measurements, demonstrating improved accuracy when applied across six oral sextants (8).

Although OPGs dominate AI-based periodontal imaging research, several studies have evaluated periapical and bitewing radiographs. Deep CNN architectures, including VGG-16, have been successfully applied to detect and classify ABL on periapical images (5). Ensembles of deep neural networks have also been used to quantify alveolar crestal height on bitewing radiographs, improving diagnostic consistency (13). Furthermore, integrating pretrained CNNs with deep neural networks and support vector machines has enabled reliable differentiation between healthy and diseased patients, even in the presence of confounding clinical factors such as missing teeth, crowns, or restorations (14).

### 3.2 Three-dimensional imaging: cone-beam computed tomography

Compared with 2D radiography, CBCT offers superior visualization of periodontal structures by minimizing anatomical superimposition and distortion. Recent AI-driven CBCT analyses have implemented multi-stage deep learning pipelines for automatic tooth segmentation and numbering, achieving accuracy rates approaching 99%. These systems enable precise identification and segmentation of multiple periodontal defect types, including total ABL, supra- and intrabony defects, endo-periodontal lesions, buccal defects, and furcation involvement. AI-driven CBCT segmentation models have reported accuracy values approaching 0.95–0.99 for tooth identification and defect classification, demonstrating high reliability in three-dimensional periodontal assessment (15). Recent advances have further demonstrated the application of AI in detecting and grading furcation involvement using CBCT imaging. Deep learning models trained on volumetric datasets have shown high accuracy in identifying the presence and severity of furcation defects, enabling improved three-dimensional characterization of periodontal destruction and facilitating more precise treatment planning. These models have demonstrated strong diagnostic performance, with accuracy values exceeding 0.90 for furcation involvement detection (16). The application of AI-based semantic segmentation to CBCT imaging represents a major advancement in periodontal diagnostics, providing detailed defect characterization and supporting improved disease staging and treatment planning. However, broader clinical adoption remains limited by cost, radiation exposure, and accessibility, underscoring the need for further validation and optimization in routine practice.

## 4 Artificial intelligence for peri-implantitis

Peri-implantitis presents significant clinical challenges due to compromised bone support, altered immune responses, and impaired wound healing, which complicate disease management and long-term prognosis. Although not all cases progress to implant loss, advanced disease may require complex therapeutic interventions aimed at infection control, defect resolution, and preservation of implant stability (17). AI–based approaches have enabled deeper characterization of host immune responses, allowing clinicians to stratify patients into high- and low-risk categories and personalize treatment strategies (18). ML - driven immune profiling has demonstrated that peri-implantitis outcomes are strongly influenced by the immune microenvironment of peri-implant tissues. Using the Fast and Robust DEconvolution of Expression Profiles (FARDEEP) algorithm, AI models have facilitated immune cell deconvolution and risk grading through transcriptomic analysis. These analyses identified immune parameters such as B-cell infiltration, macrophage polarization ratios, and inflammatory signalling pathways as key determinants of infection control, wound healing, and the likelihood of successful implant re-implantation. ML models incorporating immune profiling have demonstrated strong predictive performance, with area under the curve (AUC) values exceeding 0.85 in distinguishing high-risk peri-implantitis phenotypes. By integrating immune biomarkers with clinical data, AI-driven frameworks offer improved predictive accuracy compared with conventional diagnostic approaches alone, supporting more informed clinical decision-making in peri-implant disease management (17, 18).

## 5 Artificial intelligence to elucidate links between periodontitis and systemic diseases

Artificial intelligence and ML approaches have increasingly substantiated associations between periodontitis and a wide spectrum of systemic diseases, including cardiovascular, metabolic, infectious, and malignant conditions (18). These models integrate transcriptomic, genomic, and epigenomic datasets to identify shared molecular signatures underlying oral–systemic disease interactions. Advanced bioinformatics pipelines incorporating autoencoders and ML algorithms such as XGBoost (eXtreme Gradient Boosting) and Random Forest enable dimensionality reduction of large-scale molecular datasets and prioritize genes and microRNAs (miRNAs) most strongly associated with disease phenotypes (19). Using these approaches, investigators have identified hub genes and regulatory miRNAs linking periodontitis with systemic inflammatory and immune-mediated diseases (20). Integrative analyses have identified ANKRD29 and TDO2 as shared hub genes between periodontitis and breast cancer, suggesting immune-mediated pathways that may influence the tumor microenvironment (21). Similarly, artificial neural network–based studies have identified common hub genes linking periodontitis and COVID-19, implicating inflammasome-driven immune dysregulation as a shared pathogenic mechanism (22). ML–based molecular studies further demonstrate that keystone pathogens and pathobionts including *Fusobacterium nucleatum*, *Porphyromonas gingivalis*, *Tannerella forsythia*, *Treponema denticola*, and *Streptococcus gordonii* induce oncogenic and differentially expressed miRNAs, such as miR-126-5p, miR-103, miR-218, and miR-30d. These miRNA signatures link periodontitis to colorectal, prostate, breast, glioma, gastric, and hepatocellular carcinomas, as well as atherosclerosis and cardiovascular disease (23–28).

### 5.1 Biomarker discovery and disease mechanism elucidation

ML has substantially advanced biomarker discovery in periodontitis by enabling analysis of complex, high-dimensional molecular datasets and improving insight into host immune responses to bacterial toxins. Using deep learning and ensemble ML models, researchers can prioritize molecular features that most accurately distinguish infection status and disease severity. ML-based analyses of exosomal microRNAs derived from periodontal ligament stem cells exposed to bacterial toxins identified miR-592-5p as the strongest predictor of *T. forsythia* infection, mmu-miR-339-5p as the most influential feature associated with *F. nucleatum* infection, and 18 miRNAs strongly linked to *P. gingivalis* invasion. These miRNAs were associated with key biological pathways, including inflammasome activation and apoptotic signalling, supporting the potential of exosomal miRNAs as early diagnostic biomarkers with implications for bone regeneration (7).

### 5.2 Gingival keratinocytes and immune–inflammatory responses

Keratinized gingiva provides essential mechanical stability and microbial barrier protection. Compromises of this epithelial barrier increase susceptibility to inflammation and PD progression (29, 30). AI-driven transcriptomic analyses have enabled detailed characterization of gingival keratinocyte heterogeneity. Using autoencoder-based and ML approaches, investigators identified 28 distinct gingival keratinocyte subpopulations, revealing greater cellular diversity than previously recognized. These findings demonstrate that AI-based transcriptomic profiling can uncover novel therapeutic targets and support personalized precision medicine approaches in periodontology (31).

### 5.3 Prediction of antimicrobial resistance (AMR)

AMR is an increasing challenge in periodontal therapy as conventional antibiotics lose effectiveness against resistant oral pathogens (20). AI-based models have improved AMR prediction by integrating genomic and proteomic data to identify resistance-associated features. Interpretable ML models analyzing *T. denticola* genomic data demonstrated that AMR prediction accuracy was strongly influenced by variations in the amino acid composition of lysine (K), glutamic acid (E), and alanine (A), providing biologically meaningful insight into resistance mechanisms (19). In parallel, convolutional neural networks applied to *P. gingivalis* protein sequences enabled classification of resistant and non-resistant phenotypes and facilitated identification of potential drug target sites. These AI-driven approaches highlight the potential for rapid, sequence-based AMR diagnostics and support personalized antibiotic selection in periodontal care (32).

## 6 AI for enhancing patient adherence, dental education, and public health communication

Effective management of PD relies heavily on sustained oral hygiene practices and long-term maintenance therapy (4). AI–enabled digital health interventions to have recently emerged to improve patient adherence through real-time monitoring, feedback, and personalization. A randomized controlled clinical trial evaluated an AI-enabled multimodal-sensing toothbrush combined with targeted mobile health (mHealth) micro-messages for periodontitis management. When used adjunctively with standard periodontal therapy, this intervention significantly improved oral hygiene adherence, with measurable reductions in plaque indices and gingival inflammation scores compared to standard care by providing real-time brushing guidance and enabling remote monitoring of patient behaviours. These findings highlight the potential of AI-driven behavioral interventions to support periodontal maintenance and scalable public health strategies (33). AI has also demonstrated utility in dental education and public health communication. Natural language processing (NLP) models, including RoBERTa (Robustly Optimized BERT Pretraining Approach), have been applied to analyze public engagement with oral health content on digital platforms, facilitating identification of misinformation and assessment of public sentiment. While promising, these approaches require further refinement in model precision, dataset diversity, and class balance before widespread implementation (34). Additionally, large language models such as ChatGPT have been explored as adjunctive educational tools, demonstrating potential benefits in periodontal education while underscoring the importance of oversight, accuracy, and ethical deployment (35–37).

## 7 Challenges, limitations, and future directions

Despite the growing impact of artificial intelligence in periodontology, several barriers limit its routine clinical implementation. A major challenge is the lack of standardized, high-quality datasets, with many studies relying on publicly available data that lack comprehensive clinical metadata, including small sample sizes, or exhibit demographic bias. Manual annotation of radiographic images further introduces subjectivity and inter-examiner variability. However, variability in reported performance metrics across studies reflects heterogeneity in datasets, model architectures, and validation strategies, limiting direct comparability. The predominance of 2D radiographic imaging presents additional limitations, including anatomical superimposition and image distortion, which impair accurate assessment of periodontal parameters such as vertical bone loss. Although three-dimensional CBCT imaging overcomes many of these constraints, its broader adoption is restricted by cost, radiation exposure, and accessibility. Future research should prioritize the development of fully integrated AI-based clinical software validated in real-world settings. Such systems must accurately identify teeth across diverse clinical conditions including missing, restored, implanted, or anatomically abnormal dentition while reliably distinguishing between healthy and diseased periodontium and predicting disease risk and progression. Integration of periodontal data with patients' systemic health information may further enable prediction of related systemic conditions. Ethical considerations, including data privacy, transparency, and responsible AI governance, remain essential for successful clinical adoption.

## 8 Conclusion

AI has demonstrated high accuracy in detecting, grading, and quantifying ABL using both 2- and 3-dimensional imaging modalities, supporting improved periodontal diagnosis and treatment planning. Beyond imaging, AI-driven approaches facilitate biomarker discovery, AMR prediction, and personalized risk stratification, highlighting their potential to transform periodontal care. AI-based tools also show promise in enhancing patient education, adherence to oral hygiene practices, and professional training. However, widespread clinical adoption remains constrained by challenges related to data standardization, model interpretability, generalizability, and real-world validation. Continued research focused on integrated, patient-centered AI systems that combine clinical, radiographic, and molecular data will be essential to fully realize the potential of AI in periodontology.
